# Primary vaginal malignant melanoma: A rare case report of successful treatment with nivolumab

**DOI:** 10.1097/MD.0000000000025691

**Published:** 2021-04-30

**Authors:** Na Guo, Jiawen Zhang

**Affiliations:** a The Department of Obstetrics and Gynecology, West China Second University Hospital of Sichuan University, Chengdu, Sichuan; b Key Laboratory of Birth Defects and Related Diseases of Women and Children (Sichuan University), Ministry of Education, China.

**Keywords:** clinical features, immunotherapy, postmenopausal woman, surgery, vaginal malignant melanoma

## Abstract

**Rationale:**

Primary vaginal malignant melanoma is a sporadic and very aggressive tumor that is treated through surgery or radiotherapy combined with chemotherapy. Since most cases are diagnosed at an advanced stage, the operation range is extensive, the quality of life is poor, and the prognosis is gloomy.

**Patient concerns:**

A 58-year-old woman presented irregular water-like leukorrhea for 1 month after 6 years of menopause. Positron emission tomography-computed tomography revealed a 3.1 × 2.6 × 3.2 mass on the middle and lower part of the right vaginal wall. A gynecological examination revealed a 2 to 3 cm exophytic black mass in the lower-right part of the vaginal orifice. This mass was 2 cm from the urethral orifice. Furthermore, the mucosa of the anterior inferior vaginal wall had blackened and thickened, and there were some scattered black dots at the medial labia minora.

**Diagnosis:**

Due to the patient's symptoms with radiographic findings, the postmenopausal woman was diagnosed with primary vaginal malignant melanoma.

**Interventions:**

Surgery was done to remove the mass. The patient also underwent inguinal lymph node dissection, received immunotherapy, and was treated with nivolumab.

**Outcomes:**

After a 6-month follow-up period, the patient underwent a routine gynecological examination with negative radiological results. Moreover, no local recurrence or distant metastases were found.

**Lessons:**

This patient showed a good response to immunotherapy. With this treatment method, the prognosis is better for advanced-stage women, especially those who cannot endure the surgery. Local lesion resection and inguinal lymph node dissection combined with immunotherapy are recommended. The case reported here may help treat similar clinical cases.

## 1 Introduction

Malignant melanoma, a rare malignant tumor, accounts for approximately 1% of all tumors and can occur in many parts of the body, including the skin, mouth, nasal cavity, anal canal, esophagus, and vulva. Primary malignant melanoma originating from the female genitalia accounts for only 3% to 7% of all malignant melanomas.^[1]^ Primary vaginal malignant melanoma is a sporadic and very aggressive tumor of the female genital tract, and its prognosis is ineffectual despite the various treatment options. This is because most cases are diagnosed at an advanced stage, with a 5-year survival rate of 5% to 25%.^[2]^

Vaginal malignant melanoma is always treated with surgery or radiotherapy combined with chemotherapy, and its treatment involves radical local resection of the primary lesion tumor with lymphadenectomy. For advanced-stage women, especially those who cannot endure surgery, the prognosis is poor. Since it is rare in the clinics, there are no systematic guidelines. This report analyzes 1 case of a postmenopausal woman who suffered from primary vaginal malignant melanoma, together with a review of other relevant literature.

## 2 Case presentation

**Figure 1:**
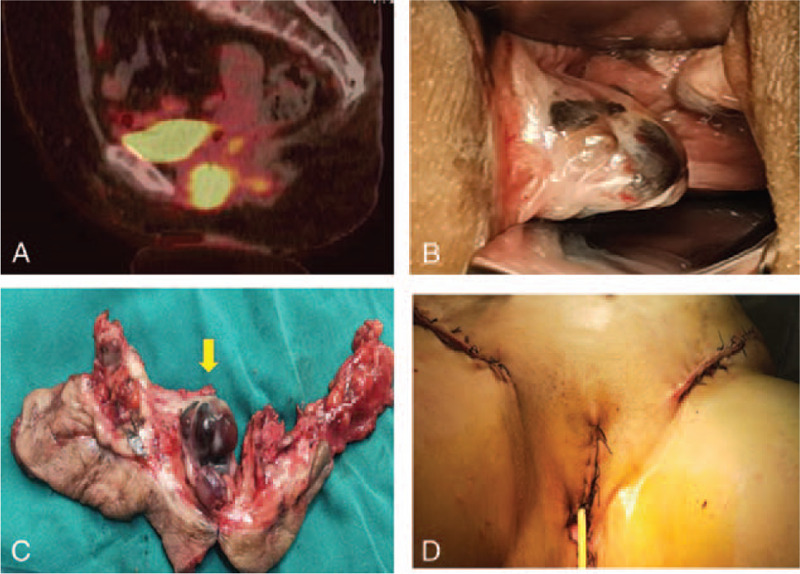
Clinical features of the patient and surgery specimen. (A) Positron emission tomography-computed tomography showing a 3.1 × 2.6 × 3.2 cm mass on the middle and lower part of the right vaginal wall. (B) Gynecological analysis showing a 2 to 3 cm exophytic black mass in the lower-right part of the vaginal orifice. (C) Surgery was performed on the lesion, which involved local excision of the tumor and inguinal lymph node dissection. (D) Surgical specimen revealing a 2.5 × 2.3 × 0.3 cm tumor in the vagina (yellow arrow).

**Figure 2:**
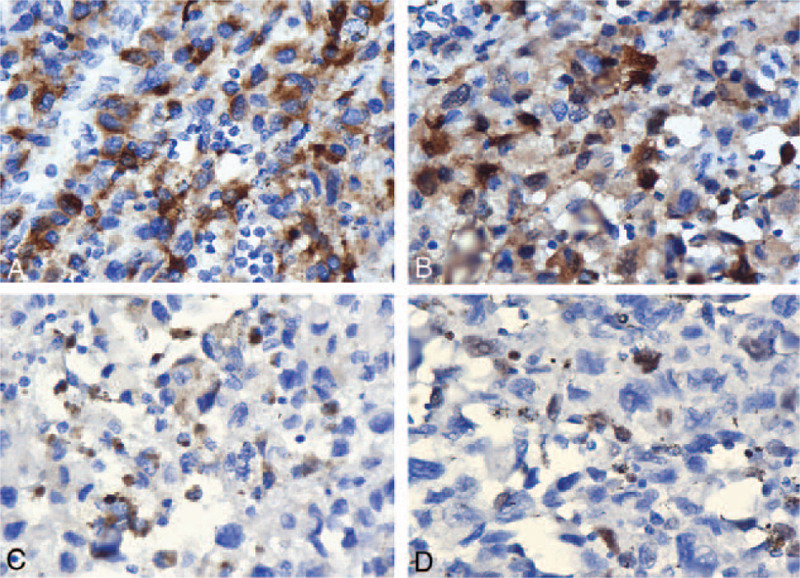
Pathological findings. (B-1) Immunohistochemical positivity for HMB-45 antibodies (magnification ×400). (B-2) Immunohistochemical positivity for S-100 antibodies (magnification ×400). (B-3) Immunohistochemical positivity for Melan-A antibodies (magnification ×400). (B-4) Immunohistochemical positivity for Ki67 antibodies (magnification ×400). HMB-45 = human melanoma black 45.

A 58-year-old woman was hospitalized with irregular water-like leukorrhea for >1 month after 6 years of menopause. She had undergone 2 normal deliveries, no history of diseases, and had been in good health before this symptom began. The patient also denied any previous medical or surgical illness. Her positron emission tomography-computed tomography investigation showed a 3.1 × 2.6 × 3.2 cm mass on the middle and lower part of the right vaginal wall (Fig. 1A). A gynecological examination revealed a 2 to 3 cm exophytic black mass in the lower-right part of the vaginal orifice, which was 2 cm from the urethral orifice. The mucosa of the anterior inferior vaginal wall had blackened and thickened, and there were some scattered black dots at the medial labia minora (Fig. 1B). According to the International Federation of Gynecology and Obstetrics (FIGO) system, the patient was clinically diagnosed with stage III disease. Surgery was performed on the lesion, which involved only local excision of the tumor and inguinal lymph node dissection (Fig. 1C). The surgical specimen revealed a 2.5 × 2.3 × 0.3 cm tumor in the vagina (Fig. 1D). The surgical specimen's pathological findings showed positivity for human melanoma black 45 (HMB-45), S-100, Melan-A, and Ki67 immunocytochemically, confirming malignant melanoma diagnosis (Fig. 2B-1–B-4). The patient had a genetic test, and the programmed cell death receptor 1 gene tested positive. After the surgery, the patient was prescribed immunotherapy, nivolumab therapy, and the programmed cell death receptor 1 monoclonal antibodies. The dose was calculated according to body weight, 3 mg/kg every 2 weeks for 12 weeks. The main adverse reaction was rashes, which disappeared after discontinuation. The monitoring aids were gynecological examination and radiological examination. After a 6-month follow-up period, the patient underwent a routine gynecological examination, with negative radiological results, and no local recurrence or distant metastases were found.

## 3 Discussion

The female reproductive system's malignant melanoma is rare, and so is the primary tumor—only a few cases have been reported (0.046/100,000).^[3]^ It can occur in many parts of the body, such as the skin, mouth, nasal cavity, anal canal, esophagus, and vulva. Virginal malignant melanoma is rare, accounting for <5% of vaginal malignant tumors, accounting for <1% of all malignancies.^[1]^ Vaginal malignant melanoma can occur at any age and any part of the vagina, mostly occurring in the distal 1/3 anterior wall of the vagina.

The main clinical features are irregular vaginal bleeding, abnormal discharge, mass, and pain. The mass can manifest as nodular, polypoid, or mushroom umbrella. An ulcer is often formed on the mass's surface, mostly blue-black or gray-black, and 10% to 23% of the masses are non-pigmented.^[4]^ Virginal malignant melanoma can be diagnosed by immunohistochemistry, S-100, HMB-45, and melanoma-associated antigen recognized by T cells (MART-1)—the latter 2 are most commonly used because of their specificity.^[5]^ Vaginal malignant melanoma is characterized by an extremely high degree of malignancy and invasiveness, high difficulty in treatment, high rate of recurrence and metastasis, and poor prognosis. Once the disease occurs, it seriously impacts the patient's body, mind, and even life. Only a few cases have been reported worldwide, so there are no clear diagnosis and treatment guidelines.

Vaginal malignant melanoma is always treated with surgery or radiotherapy combined with chemotherapy. Because of its insensitivity to chemoradiotherapy, surgery is the first choice for malignant melanoma treatment, including local extended resection and radical excision. Pelvic organ removal and lymphadenectomy radical excision surgery are often the first options. Since most cases are diagnosed at an advanced stage, the operation range is large, the quality of life poor, its recurrence rate high, and the prognosis very poor.^[6]^

Recent studies have shown that immunotherapy with checkpoint inhibitors and targeted therapy has greatly improved the prognosis.^[1]^ Patients with poor autoimmune function or immune deficiency have a higher risk of suffering from vaginal malignant melanoma than normal immune people. The disease also has certain heredity; therefore, immunotherapy is a new treatment strategy for primary vaginal malignant melanoma. National Comprehensive Cancer Network guidelines reported that the first-line adjuvant treatment for vaginal malignant melanoma was immunotherapy. Immunotherapy based on Programmed death-1 (PD-1) receptor monoclonal antibodies has made progress in treating vaginal malignant melanoma; the effective treatment rate of PD-1 is >50% for patients, and progression-free survival is nearly 1 year.^[7]^ Nivolumab is a monoclonal antibody that blocks PD-1 from binding to its ligands and has been approved to increase the overall survival and progression-free survival compared with chemotherapy.^[8]^ Extant literature has reported a total of 805 vaginal malignant melanoma cases. The first treatment was surgery, surgery combined with adjuvant radiotherapy, adjuvant immunotherapy, or adjuvant chemotherapy. Radiotherapy or immunotherapy was the most common treatment, occurring in 61% of the cases. The mean duration of recurrence-free and overall survival were 16.4 and 22.2 months, respectively.^[9]^ Some researchers have reported that anti-PD-1 therapy has a more favorable benefit–risk ratio than other adjurant therapies and should be used preferentially.^[10]^ Another study reported that anti-PD-1 treatment is related to more improved outcomes than immune therapy regarding treating women lower genital tract melanoma.^[11]^

In the present case, the surgical specimen's pathological findings were positive for HMB-45, S-100, and Melan-A immunocytochemically, so the examination results qualified for malignant melanoma. Surgery was performed on the lesion to remove the mass and dissect the inguinal lymph node. After that, the patient received immunotherapy and was treated with nivolumab. After a 6-months follow-up period, she underwent a regular gynecological examination, with negative radiological results, and no local recurrence or distant metastases were found. Therefore, it was concluded that the disease responded to immunotherapy. Thus, we suggest that for women with primary vaginal malignant melanoma—especially those who cannot endure major surgery—only a local excision of the tumor, followed by immunotherapy, can help treat primary vaginal malignant melanoma.

This patient showed good response to immunotherapy. Prognosis is better for advanced-stage women, especially those who cannot endure the surgery. Such cases require local lesion resection and inguinal lymph node dissection, followed by immunotherapy.

## Author contributions

**Data curation:** Na Guo.

**Funding acquisition:** Na Guo.

**Investigation:** Jiawen Zhang.

**Methodology:** Jiawen Zhang.

**Writing – original draft:** Na Guo.

**Writing – review & editing:** Jiawen Zhang.
